# Supplementary material for: A Mixture of Dietary Plant Sterols at Nutritional Relevant Serum Concentration Inhibits Extrinsic Pathway of Eryptosis Induced by Cigarette Smoke Extract
Source: Int J Mol Sci. 2023 Jan 9;24(2):1264. doi: 10.3390/ijms24021264 (PMC9861561; doi:10.3390/ijms24021264)
Supplement: Supplementary file 1 [file ijms-24-01264-s001.zip › ijms-2134177-supplementary.pdf]

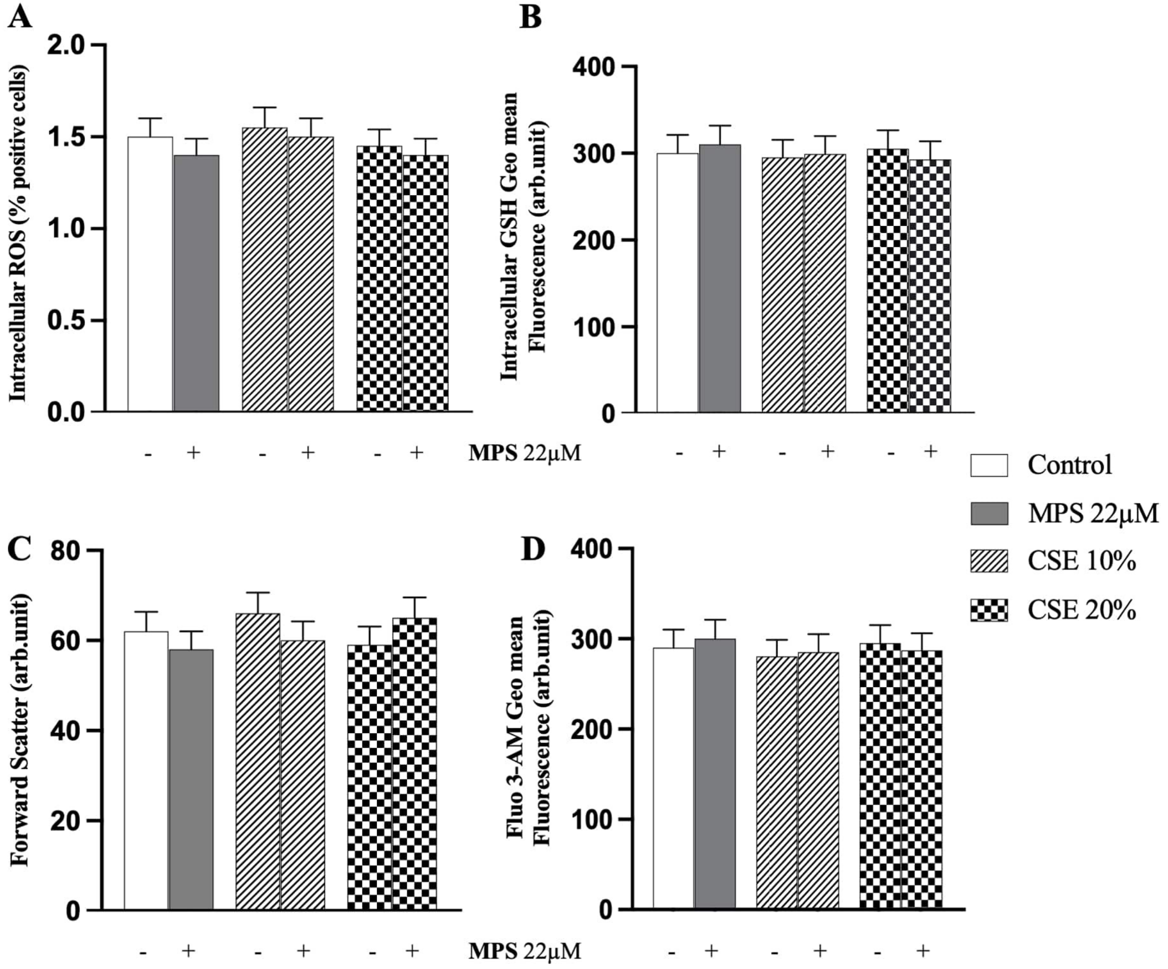

Figure S1. CSE does not induce variations of ROS, GSH, Ca^{2+} and FSC. *(**A**)* Intracellular ROS, *(**B**)* intracellular GSH, *(**C**)* variation of cell volume measured by FSC and *(**D**)* intracellular Ca^{2+} levels in 10% or 20% CSE-treated RBCs incubated for 4 h in the absence or in co-treatment with MPS 22 μM measured by flow cytometry. Protocol of measurement to chapters 4.4, 4.5, 4.6 and 4.7. RBCs incubated with Ringer solution were used as control. Values are means ± SD of (n=6) experiments carried out in triplicate.
